# Benchmarking deep learning for automated peak detection on GIWAXS data

**DOI:** 10.1107/S1600576725000974

**Published:** 2025-02-28

**Authors:** Constantin Völter, Vladimir Starostin, Dmitry Lapkin, Valentin Munteanu, Mikhail Romodin, Maik Hylinski, Alexander Gerlach, Alexander Hinderhofer, Frank Schreiber

**Affiliations:** a Institute of Applied Physics – University of Tübingen Auf der Morgenstelle 10 72076Tübingen Germany; b Cluster of Excellence ‘Machine learning – new perspectives for science’University of Tübingen Maria-von-Linden-Straße 6 72076Tübingen Germany; Australian Synchrotron, ANSTO, Australia

**Keywords:** deep learning, grazing-incidence wide-angle X-ray scattering, GIWAXS, peak detection, convolutional neural networks, Faster R-CNN

## Abstract

Recent advancements in X-ray sources and detectors have dramatically increased data generation, leading to a greater demand for automated data processing. This is particularly relevant for real-time grazing-incidence wide-angle X-ray scattering (GIWAXS) experiments which can produce hundreds of thousands of diffraction images in a single day at a synchrotron beamline. Deep learning (DL)-based peak-detection techniques are becoming prominent in this field, but rigorous benchmarking is essential to evaluate their reliability, identify potential problems, explore avenues for improvement and build confidence among researchers for seamless integration into their workflows. However, the systematic evaluation of these techniques has been hampered by the lack of annotated GIWAXS datasets, standardized metrics and baseline models. To address these challenges, we introduce a comprehensive framework comprising an annotated experimental dataset, physics-informed metrics adapted to the GIWAXS geometry and a competitive baseline – a classical, non-DL peak-detection algorithm optimized on our dataset. Furthermore, we apply our framework to benchmark a recent DL solution trained on simulated data and discover its superior performance compared with our baseline. This analysis not only highlights the effectiveness of DL methods for identifying diffraction peaks but also provides insights for further development of these solutions.

## Abstract 2

Automated data processing is is needed to handle the vast amount of X-ray scattering data generated during grazing-incidence wide-angle X-ray scattering (GIWAXS) experiments. This publication provides a metric, dataset and baseline to assess the quality of a recent deep learning peak-detection method.

## 1. Introduction

The recent development of next-generation X-ray sources such as diffraction-limited synchrotrons and X-ray free-electron lasers has led to a drastic increase in the amount of data produced (Ludwig, 2019 ▸; Dong *et al.*, 2021 ▸; Qin & Bauer, 2010 ▸; Helliwell, 2019 ▸; Helliwell *et al.*, 2017 ▸). Furthermore, the latest generations of detectors enable experiments with high resolution in both the spatial and the temporal dimensions (Wang *et al.*, 2018 ▸; Heiss, 2019 ▸). The volume of data opens up new possibilities but makes manual data analysis infeasible. Large datasets demand the use of automated solutions for processing and analysis, while deep learning (DL) receives significant attention in the field (Hinderhofer *et al.*, 2023 ▸; Karniadakis *et al.*, 2021 ▸). DL stands out with its ability to comprehend intricate relationships, its capacity for generalization and its ability to handle vast amounts of data (Pithan *et al.*, 2023 ▸; Starostin *et al.*, 2022*b* ▸; Greco *et al.*, 2022 ▸; Hinderhofer *et al.*, 2023 ▸; Guo *et al.*, 2016 ▸; Du *et al.*, 2016 ▸). A notable application of DL in this respect is the detection of Bragg peaks in X-ray scattering data, as demonstrated in the literature (Sullivan *et al.*, 2019 ▸; Liu *et al.*, 2022 ▸; Hadian-Jazi *et al.*, 2021 ▸; Yin *et al.*, 2022 ▸). The present paper evaluates the per­formance of the DL-based Bragg-peak-detection algorithm proposed by Starostin *et al.* (2022*a* ▸) and compares it with a conventional region-growing peak-finding algorithm. More specifically, these algorithms are applied to grazing-incidence wide-angle X-ray scattering (GIWAXS) data, which can be used to extract a wealth of structural information from thin films on the atomic scale (Feidenhans’l, 1989 ▸; Banerjee *et al.*, 2020 ▸; Robinson & Tweet, 1992 ▸). The ability to determine the arrangement and periodicity of crystal structures and their preferred orientations makes it indispensable in materials research and development (Steele *et al.*, 2023 ▸; Hu *et al.*, 2017 ▸; Brinkmann *et al.*, 2022 ▸). To extract this information, a key step is to accurately determine the positions, widths and intensities of Bragg peaks. However, expert knowledge is required to effectively fit the peaks due to the presence of diffuse background, experimental artefacts, scattering from the substrate and other environmental factors (Pauw, 2013 ▸). With hundreds of thousands of images captured per beam time, this laborious and time-consuming manual process becomes a bottleneck and presents a compelling case for implementing automated data analysis. The work by Starostin *et al.* (2022*a* ▸) introduces such an automated peak detection using a two-stage object detection model. In the present study, we provide a test dataset, metrics and a baseline to evaluate the suggested approach. For our test dataset, we collected a diverse set of labelled GIWAXS data from perovskite films. This dataset encompasses a range of background signals, Debye–Scherrer rings with diverse widths and intensities, and Bragg peaks of varying shapes, intensities and proximities to one another. We find that traditional object detection metrics fail to capture critical aspects of peak-detection quality, which are essential for the subsequent analysis. As a result, we propose a physics-informed metric that prioritizes the radial over the azimuthal direction. For context, we implemented a baseline using a conventional region-growing algorithm (see *Appendix B* in the supporting information) commonly used for peak detection (Guo *et al.*, 2019 ▸). The introduced dataset, metrics and baseline were used for a comprehensive evaluation of the DL solution.

## 2. Dataset

### 2.1. Dataset

To facilitate a comparative analysis of peak detection in GIWAXS patterns, we curated a dataset collected during different steps of crystallization and annealing of various perovskite thin films prepared by spin coating as detailed by Kneschaurek *et al.* (2023 ▸). This includes various 2D and 3D perovskites with varying cations (caesium, methylammonium, formamidinium or their mixtures), anions (iodine, bromine) and spacer molecules [phenethylammonium, phenylene­dimethylammonium, pentafluoro­phenylethylammonium or (1-adamantyl)methylammonium]. The selected patterns con­tain features corresponding to the final perovskite structures as well as different intermediate products (*e.g.* complexes with solvents) and precursors (lead iodide, lead bromide *etc.*). A glass slide covered with indium tin oxide (ITO) or fluorine-doped tin oxide (FTO) and (optionally) mesoporous titanium oxide layers was used as a substrate. A full list of the selected compositions and structures is provided in the supporting information.

The dataset was measured at two experimental facilities: the P08 beamline at the Deutsches Elektronen-Synchrotron (DESY) and the ID10 beamline at the European Synchrotron Radiation Facility (ESRF). The X-ray energy varied between 18 and 22 keV, and the incidence angle varied between 0.1 and 0.5° (*i.e.* below and above the critical angle). Furthermore, our dataset features patterns that exhibit different resolutions, since they were measured using a PerkinElmer XRD 1621 detector with 2048 × 2048 pixels with a pixel size of 200 µm at the P08 beamline and a Pilatus 300K detector with 487 × 619 pixels with a pixel size of 172 µm at beamline ID10. For the latter, we merged several images of the same sample at different detector positions to fill the detector gaps.

### 2.2. Preprocessing

**Figure 1:**
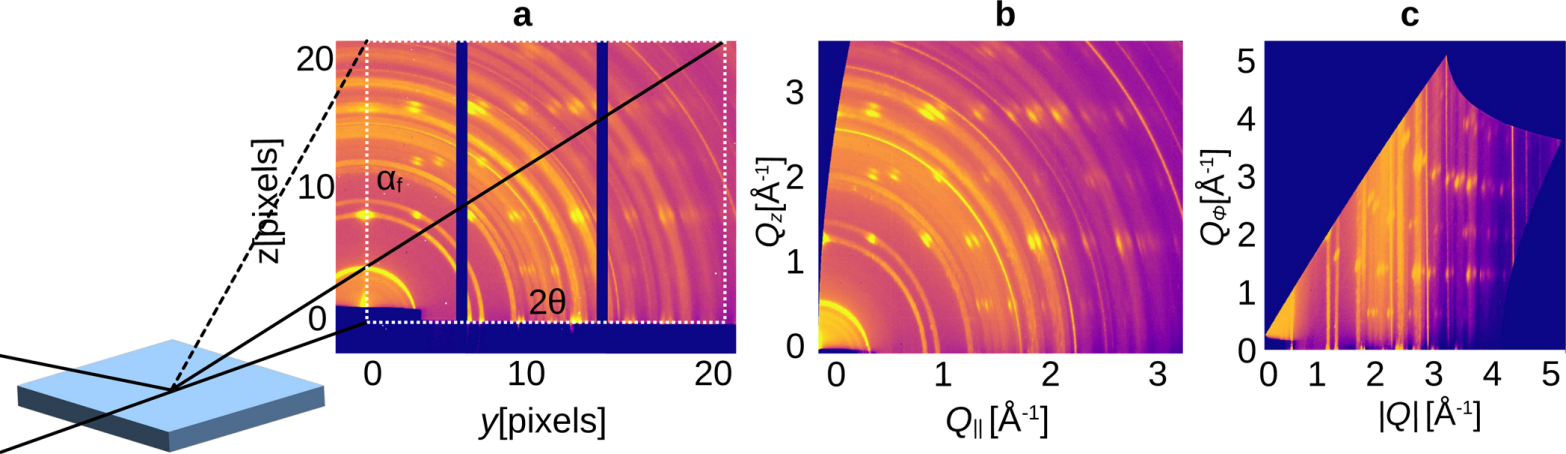
Geometry of GIWAXS experiments. Following the measurement, two acquired images are merged to remove the detector gaps and are subsequently converted from (*a*) detector coordinates to (*b*) reciprocal space coordinates and finally to (*c*) polar coordinates. For peak detection, the contrast is enhanced by CLAHE as described in Section 2.2. All shown images are already contrast-enhanced for visualization.

The raw data in detector coordinates contain Debye–Scherrer rings, in the form of arcs and arc segments, and Bragg peaks. This geometric configuration poses a challenge for the majority of computer vision algorithms, as many object detection techniques rely on rectangular bounding boxes and rectangular-shaped image filters. Consequently, we transform the GIWAXS images into a more computer-vision-compatible representation, utilizing a two-step image transformation. The GIWAXS image is first mapped from detector coordinates to sample-associated reciprocal space (*Q*_||_, *Q_z_*) (Als-Nielsen & McMorrow, 2011 ▸), and then converted to polar coordinates (|*Q*|, ϕ) defined as 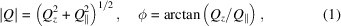
where |*Q*| is the radial coordinate and ϕ is the azimuthal angle counted from the sample horizon. For the region-growing approach, we discovered better performance with a conversion that does not distort the length of the peaks along the azimuthal direction. Instead, it focuses on the conservation of the azimuthal width of each peak, as shown in Fig. 1 ▸. We therefore chose the following conversion to (|*Q*|, *Q*_ϕ_) defined as
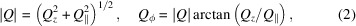
where *Q*_ϕ_ is the distance along an azimuthal arc of radius |*Q*| counted from the sample horizon. Note that *Q*_ϕ_ is not an actual scattering vector. The resulting images have a resolution of 1024 × 512. We provide an HDF5 file with the GIWAXS images in reciprocal space, along with a conversion script, on Zenodo (Völter *et al.*, 2024 ▸).

Depending on the specific measurement conditions and model of X-ray detector employed, the images exhibit unevenly distributed intensity levels, which can make it challenging to identify the peaks. To address this, we employed a contrast-limited adaptive histogram equalization (CLAHE) (Ketcham *et al.*, 1974 ▸), which distributes the intensities evenly across the histogram. Note that the histogram equalization is applied exclusively for peak detection. Further analysis and fitting of the Bragg peaks is performed using the original data.

### 2.3. Annotation

**Figure 2:**
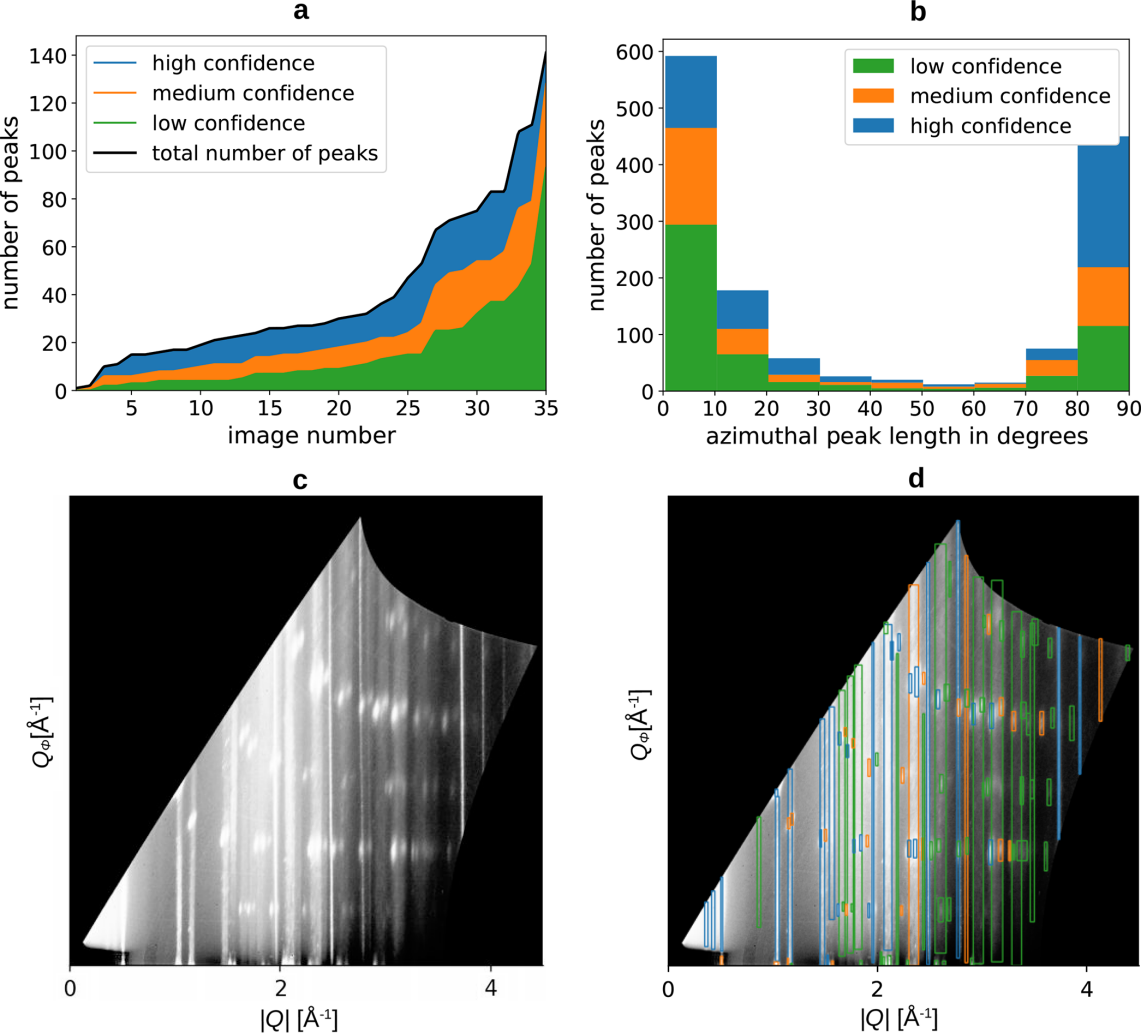
(*a*) Number of peaks per image in the evaluation dataset, sorted by the number of peaks. (*b*) Azimuthal length of peaks in the evaluation dataset. (*c*) and (*d*) Exemplary GIWAXS images from the dataset with labelled peaks. The colour is chosen according to the confidence level; the contrast is enhanced by CLAHE as described in Section 3.1.

To assess the precision of automated peak fitting, we manually annotated the radial and azimuthal positions of each Bragg peak in the described patterns with a bounding box. In the radial dimension, we separately fitted each peak with a Gaussian function on top of a linear background: 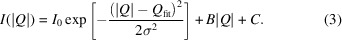
For the corresponding radial box width, we used the full width at half-maximum 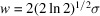 of the Gaussian function. In contrast, the peaks exhibit different shapes in the azimuthal direction, ranging from homogeneous segments of Debye–Scherrer rings to sharp and isolated Bragg peaks. This makes it impossible to use a single function for fitting. Consequently, we manually establish the boundaries for each peak in the azimuthal direction. We employ three confidence levels to characterize the prominence of the peaks. Bright peaks exhibiting a distinct Gaussian shape are assigned a high confidence rating, while peaks that are more challenging to discern receive a medium confidence level. The low-confidence category encompasses peaks that are scarcely visible and pose considerable difficulty in detection. They are barely above the background level or are covered by more intense neighbouring peaks. Fig. 2 ▸(*a*) shows both the peak count and the distribution of confidence levels of the 1448 peaks in the dataset. Fig. 2 ▸(*b*) shows the distribution of the azimuthal lengths of the peaks attributed to different confidence levels.

## 3. Automatic peak detection

### 3.1. Deep learning

The work by Starostin *et al.* (2022*a* ▸) introduces a two-stage object detection model for peak detection and algorithms to further process the information obtained. We focus on the peak detection which employs a modified faster region-based convolutional neural network (Faster R-CNN) (Ren *et al.*, 2015 ▸) tailored to the GIWAXS geometry.

Ren *et al.* (2015 ▸) used convolutional neural networks (CNNs) to create feature maps from the given input image. These feature maps are abstract representations of the input image generated by a dot product operation with a convolutional kernel (LeCun *et al.*, 2015 ▸). We adapted the kernels of the CNN to be asymmetric, which reduces the image size mostly in the vertical direction. This elongated shape is well optimized for Debye–Scherrer rings and Bragg peaks, which are typically broadened in the azimuthal direction due to the sample mosaicity.

**Figure 3:**
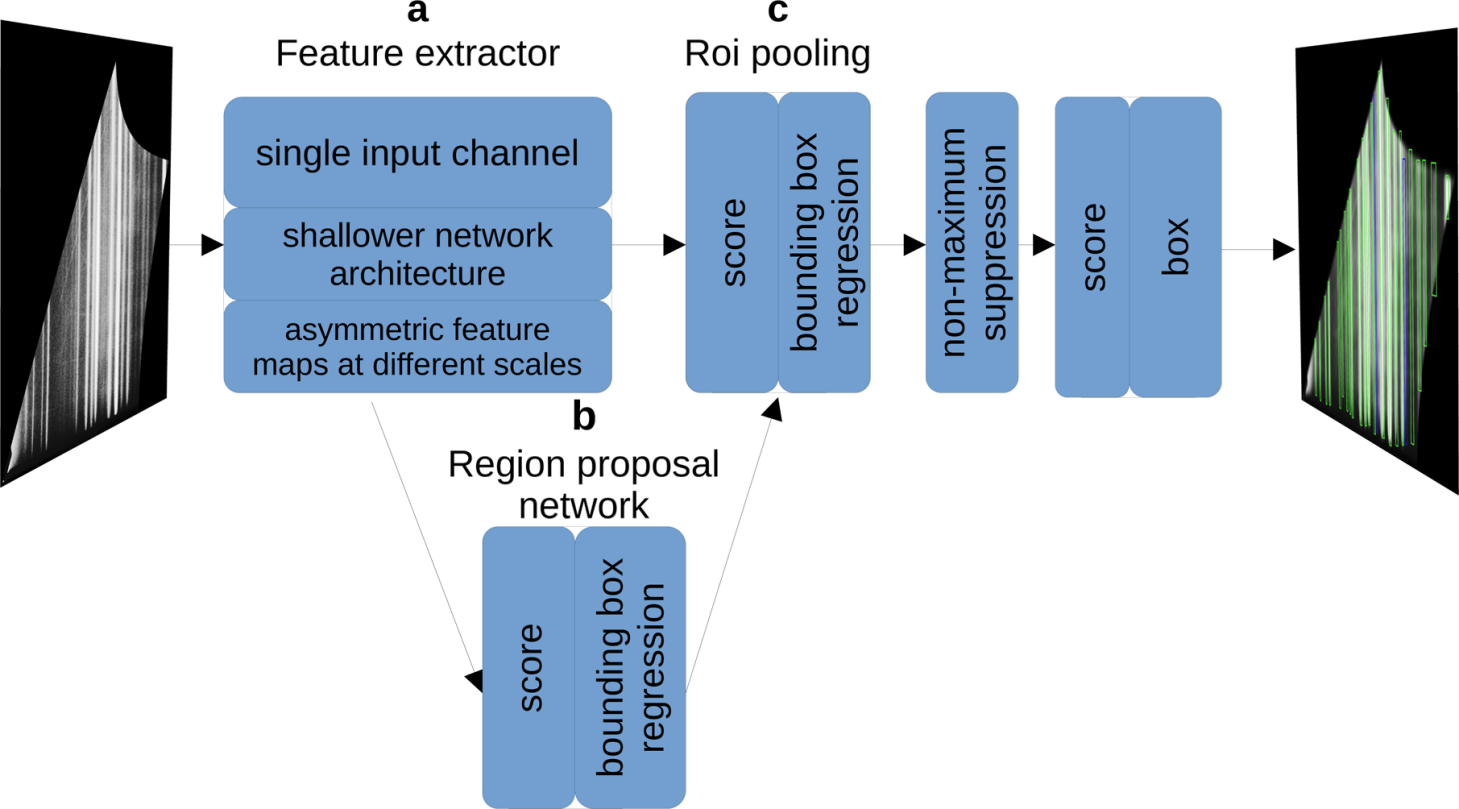
Overview of the modified Faster R-CNN structure. The feature extractor in (*a*) produces feature maps of different sizes and hands them on to the RPN in (*b*). This layer extracts regions of interest (Rois) from the feature maps at different scales. The pooling layer in (*c*) aligns the proposed boxes from (*b*) and the largest feature map in (*a*). Non-maximum suppression eliminates overlapping boxes, resulting in the final predicted boxes together with their confidence scores.

For the second detection stage, the Faster R-CNN architecture uses feature maps of multiple scales. We modified this behaviour by including only the largest feature map. This prevents the network from confusing several distinct segments with one pronounced segment. A key part of the Faster R-CNN is the region-proposal network (RPN). It slides over the feature maps and creates proposals for potential objects. We pad the target boxes for the RPN such that the proposed regions contain more background area, which provides more context for the second detection stage. Furthermore, the RPN uses a reduced number of 64 channels. This shallower network architecture was found to be sufficient for this task. Additional customizations leverage the grey-scale nature of the images by using a single colour input channel. Since Bragg peaks are the only relevant class, the classifier component of the network is eliminated. These modifications lead to a substantial increase in processing speed while maintaining a high degree of accuracy. Fig. 3 ▸ shows the general structure of the Faster R-CNN and highlights the modifications of Starostin *et al.* (2022*a* ▸).

### 3.2. Region-growing approach

**Figure 4:**
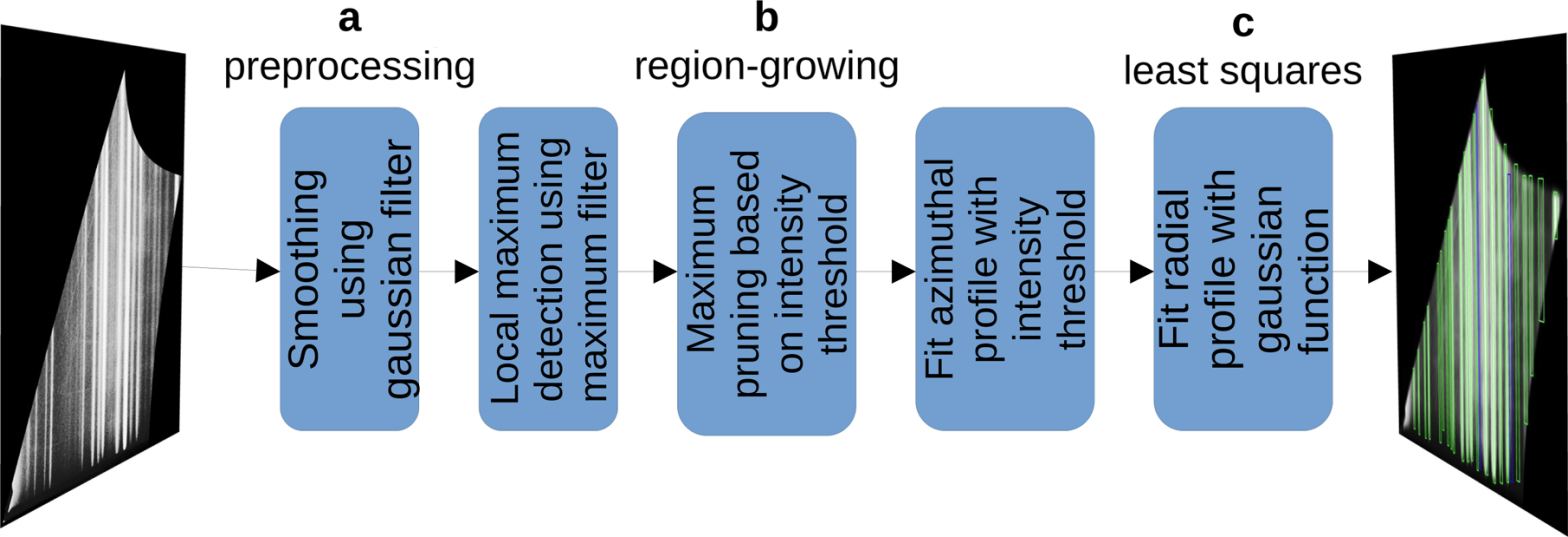
Peak-detection pipeline using a region-growing algorithm. (*a*) Image is smoothed using a 3 × 25 pixel Gaussian filter and a 3 × 3 pixel box filter. (*b*) The region-growing implementation detects peaks and fits their profile in the azimuthal direction. (*c*) A least-squares algorithm fits the radial profile using a Gaussian function with a linear background.

To compare the performance of the DL approach against a standard solution, we developed a conventional algorithm based on a region-growing approach. Though tailored for the specific analysis of GIWAXS patterns, this algorithm serves as an exemplary demonstration of the differences between conventional and DL approaches. To provide an overview of the algorithm, Fig. 4 ▸ outlines the workflow.

Since this peak-detection method is primarily based on the difference in brightness between the peaks and the background, it is essential to smooth the background while keeping the shape of the original peaks [Fig. 4 ▸(*a*)]. In our experiments, we discovered that the Gaussian and box filters produce an optimal blurred image for further processing. This denoising process significantly impacts the peak intensity and can noticeably shift the peak positions. Consequently, accurate peak positions must be determined through a peak fitting on the original, non-denoised image data.

**Figure 5:**
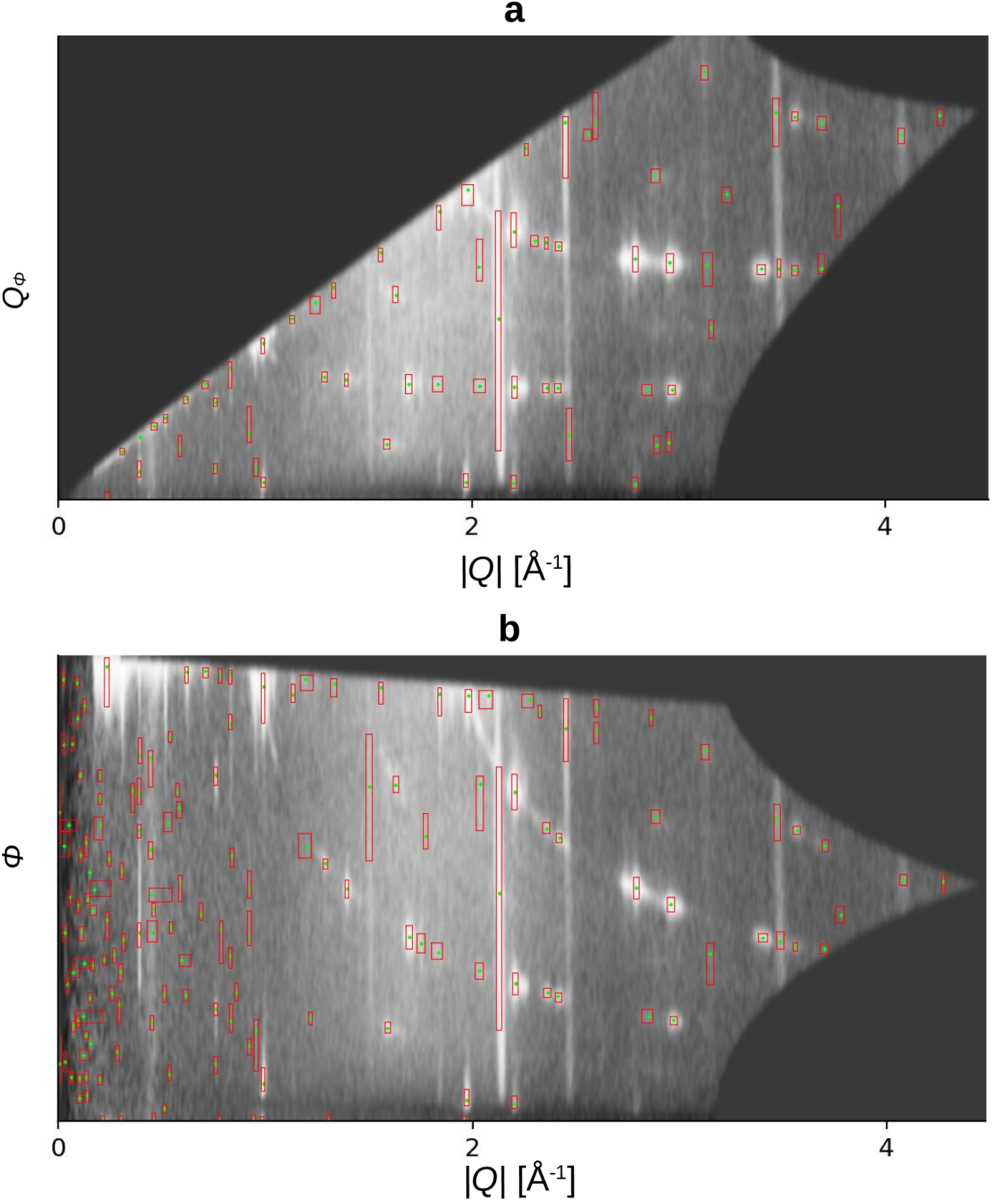
Detection results of the region-growing algorithm on the (*a*) undistorted and (*b*) distorted polar geometry. The red boxes and green points show a detected peak.

As explained in Section 2.2, we used a different conversion to polar coordinates. This geometry is chosen because the distortion along the azimuthal direction presents a significant challenge for the region-growing algorithm (see Fig. 5 ▸). We find the geometry in Fig. 5 ▸(*b*) unsuitable for the region-growing algorithm due to the high number of false detections in the low-|*Q*| range. We believe this is a result of the blurring filters applied after the interpolation. Though noise of only a few pixels can be blurred, noisy pixels in the low-|*Q*| region can expand in the polar coordinates and cannot be smoothed by the blurring. As a consequence, the region-growing algorithm detects them as maxima.

In the next step, the preprocessed image is utilized to detect intensity maxima in a two-way approach. Initially, local maxima are detected [Fig. 4 ▸(*b*)], and subsequently, the most prominent ones are selected among them [Fig. 4 ▸(*b*)]. We employ the Python implementation of Waithe (2023 ▸) which was initially proposed by Rueden *et al.* (2017 ▸).

To identify the local maxima, the algorithm employs a 3 × 3 pixel maximum filter, comparing the highest value within a 3 × 3 pixel neighbourhood with the corresponding values in the unfiltered image. Locations with identical values are identified as local maxima. Once the local maxima have been identified, the algorithm finds the global maxima across the entire image. This is achieved by growing the region around a maximum until an intensity threshold of 14 is met. This threshold corresponds to 5.5% of the absolute image brightness. The algorithm then combines the local maxima of a region into a single maximum. We determined the tunable parameters shown in Table 1 ▸ through systematic experimentation to achieve the highest recall values on the dataset described in Section 2.

To compare with the work of Starostin *et al.* (2022*a* ▸), relying solely on the approximate peak position is not sufficient. In the radial direction, we employed the least-squares method to fit each peak with a Gaussian function on top of a linear background [Fig. 4 ▸(*c*)]. The boundary in the azimuthal direction is determined by the region-growing algorithm using the intensity threshold of 14 [Fig. 4 ▸(*b*)].

## 4. Metrics

To evaluate the effectiveness of the two peak-detection methods, it is necessary to employ a metric designed specifically for Bragg peak detection. Hence, we will briefly examine metrics from related areas and tailor them to our specific use case. Hinderhofer *et al.* (2023 ▸) classify the task of Bragg peak detection as an object detection problem in the context of computer vision. This categorization is advantageous as it provides access to a range of well established metrics for evaluating and quantifying performance (Padilla *et al.*, 2020 ▸). One commonly used metric is the average precision, which assesses the accuracy by estimating the area under the precision–recall curve. The fundamental concept revolves around determining the intersection over union (IoU) criterion between the predicted box and the ground truth box: 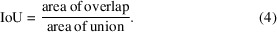
The identified bounding boxes are subsequently categorized as true positives, false positives or false negatives on the basis of the selected threshold for the IoU criterion (see Table 2 ▸).

The precision *P* and recall *R* are then determined by calculating the ratio of correct positive detections over all detections and all ground truths, as
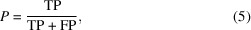

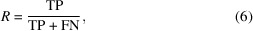
where TP, FP and FN are the numbers of true positives, false positives and false negatives, respectively. The precision represents the algorithm’s ability to accurately identify relevant objects among the predicted instances. On the other hand, the recall quantifies the algorithm’s capacity to identify all of the given ground truth instances. Different confidence scores of the model result in different trade-offs between precision and recall points, which form a precision–recall curve *P*(*R*). A high area under the curve indicates that many objects are recalled with high-quality intersections. Instead of integrating the whole area, a traditional approach is to interpolate the shape at 11 precision values (Schütze *et al.*, 2008 ▸). The result is the average precision 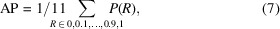
where *P*(*R*) is the precision value *P* at the corresponding recall value *R*.

### 4.1. GIWAXS-specific metrics

While equation (7) is a commonly used metric for standard object detection tasks, we have identified the need for some adjustments to suit our specific peak-detection methods better. The rationale stems from the features typically observed in GIWAXS patterns. The GIWAXS patterns contain Bragg peaks with a small, sharp Gaussian shape in the radial direction, whereas the azimuthal width is typically substantially larger. For determining the crystalline structure, the radial peak position is more important and requires a significantly higher level of precision in the radial direction compared with the azimuthal one. The IoU as a criterion of intersection is only partially suitable for this purpose since it treats both directions equally. Furthermore, the average precision is a single number to determine the quantity of intersections and only partially takes into account the quality of intersections. Therefore, we propose splitting the metric as explained in the following. The accurate determination of intensity requires a robust fit in both the radial and the azimuthal angles. Therefore, the average IoU (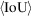) is well suited for this specific purpose. To place additional emphasis on the radial direction, we propose to compute the IoU based only on the overlap in the radial direction (IoU_*r*_). The average IoU in the radial direction 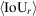 reliably measures the quality of intersection among detected peaks. Given a specific IoU_*r*_ threshold, the average precision can be determined using the *P_r_* and *R_r_* values:
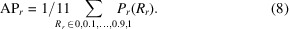
We adhere to the conventional 11-point interpolation because of its clear computational advantage in estimating the shape of the entire area under the curve.

Given the significance of the peak positions in the radial direction |*Q*|, the average distance between the detected |*Q*_detected_| and ground truth |*Q*_truth_| peak positions –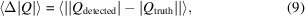
where the averaging is performed over all detected peaks – is of particular interest.

## 5. Results and discussion

We applied the peak-detection methods described in Section 3 with the composed dataset described in Section 2 and evaluated the results using the proposed metrics. For the classification of peaks as TPs, a minimum IoU_*r*_ value of 0.1 was used. Since the region-growing approach does not have a confidence score, we did not calculate the average precision but used the *P_r_* of equation (5) based on a minimum IoU_*r*_ value of 0.1. The *P_r_* value of the modified Faster R-CNN is calculated for a minimum confidence score of 0.1 and an IoU_*r*_ value of 0.1. The results are summarized in Table 3 ▸. We observe that the DL approach outperforms the region-growing algorithm in almost all metrics.

Using the region-growing approach as a reference, the ‘Recall’ metric demonstrates that the Faster R-CNN approach detects more peaks in the high and medium confidence levels, whereas the region-growing approach has a slightly higher value for the low-confidence peaks. The detection of weaker peaks poses a challenge for both methods. Although the AP_*r*_ cannot be determined for the region-growing algorithm, the modified Faster R-CNN shows a promising value of 70% on experimental data. Note that the DL model is trained and fine-tuned on simulations, for which it reaches an AP_*r*_ value of 99% (Starostin *et al.*, 2022*a* ▸). This gap could be reduced by including annotated, experimental data into the fine-tuning process. When contrasted with the region-growing approach, the Faster R-CNN shows a significantly higher *P_r_* of 87% as opposed to 62%. Although both approaches have the same IoU_*r*_ in the radial direction, the average peak distance 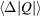 shows that DL detects peak centres more precisely. The IoU shows the same behaviour in the azimuthal direction: the DL approach demonstrates a superior fit.

**Figure 6:**
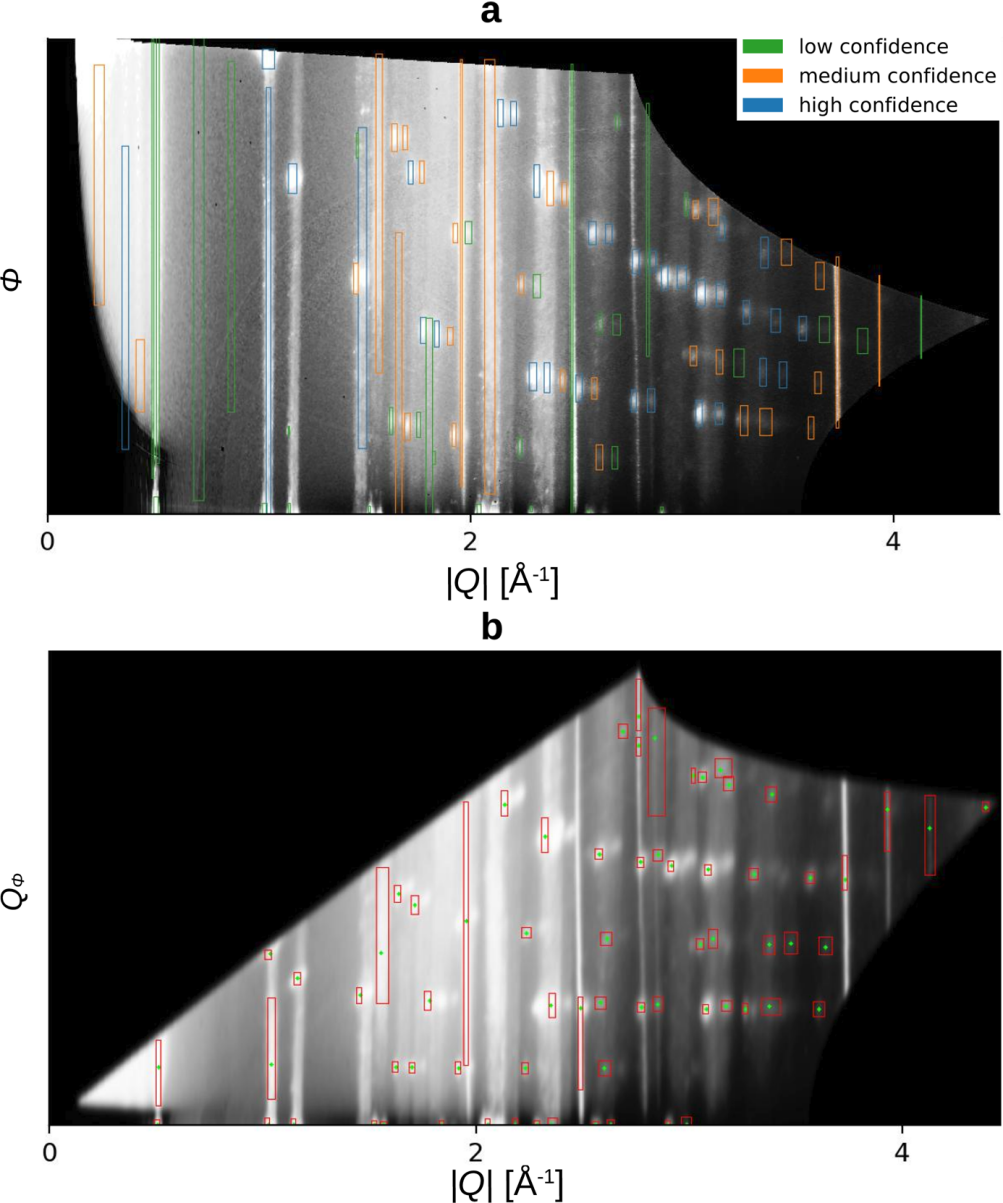
Peak-detection results from (*a*) the modified Faster R-CNN and (*b*) the region-growing approach. The colours in (*a*) are chosen according to the confidence score of the DL-model, not the confidence score of the ground truth labels. The different geometries are explained in Section 2.2, the blurring of (*b*) in Section 3.2. (*b*) Detected maxima are shown as green points. The red bounding boxes are the result of the fitting explained in Section 3.2.

Fig. 6 ▸ is an example from the evaluated dataset, visually confirming the results in Table 3 ▸. It demonstrates that the region-growing approach has a lower recall for high- and medium-confidence peaks. The 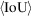 is lower for the region-growing approach due to significant issues with azimuthally extended peaks, especially with Debye–Scherrer rings. In contrast, the radial intersection over union (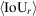) performs equally well for both algorithms. Evaluating 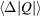 visually is challenging without seeing the ground-truth boxes. All evaluated GIWAXS images, including the marked detection results, are available on Zenodo (Völter *et al.*, 2024 ▸).

### 5.1. Recall

We consider Bragg peak detection as effective if it achieves the highest possible recall value. As we can see in Table 3 ▸, both algorithms excel at detecting the brighter, high-confidence peaks, with recall values of more than 88%, but have problems detecting the less intense low-confidence peaks, showing results of 55 and 60%. We explain these results by examining how the algorithms extract relevant features.

In our specific case of detecting Bragg peaks, the region-growing algorithm uses the intensity of the peaks as a feature. The expert must determine a suitable intensity threshold to distinguish meaningful peaks from noise and background artefacts. Unfortunately, this feature is highly vulnerable to background scattering; the intensity as a characteristic alone may not be sufficient to achieve excellent results. This task requires more sophisticated features to enhance the performance further.

In contrast, a DL model possesses an intrinsic ability to detect features from a dataset. The DL model employed by Starostin *et al.* (2022*a* ▸) is trained on a simulated dataset with varying peak intensities and captures these features by employing millions of training parameters. Nonetheless, the recall metrics in Table 3 ▸ indicate that the model struggles to detect less prominent peaks. Hence, enhancing the performance of feature detection could be realized through either a broader range of simulations or modifications to the model that enable the identification of peaks with low intensity.

Bragg peaks in GIWAXS data can manifest at arbitrary spatial positions, including border regions of the image. It is not uncommon for peaks to be located close to each other or even be situated on top of the Debye–Scherrer rings. Ensuring a high recall in peak detection demands a robust algorithm capable of handling these characteristics.

Both of the discussed peak-detection methods demonstrate the capability to identify Bragg peaks at various positions. The approach to GIWAXS peak detection introduced by Starostin *et al.* (2022*a* ▸) uses a diverse training dataset encompassing peaks with multiple spatial arrangements and therefore enables the identification of Bragg peaks across the entire image. Similarly, the region-growing approach detects local maxima across the entire image, highlighting its translational equivariance in peak detection.

We observe that Bragg peaks often appear very close to each other, which can result in missing out on individual peaks or mistakenly combining two into one. To achieve a high recall value, we require an algorithm that can accurately detect even these closely spaced peaks. However, the non-maximum suppression mechanism inherent in the Faster R-CNN architecture introduces challenges when dealing with these adjacent peaks. If a neighbouring peak holds lower confidence compared with another peak but significantly overlaps with it, the non-maximum suppression may erroneously suppress it (Bodla *et al.*, 2017 ▸). Furthermore, the non-maximum suppression mechanism is not equipped to handle instances where objects are nested, as exemplified by a Bragg peak atop a Debye–Scherrer ring. In contrast, the region-growing approach distinguishes peaks from one another on the basis of intensity thresholds. This approach performs effectively for prominent peaks, but it encounters challenges when dealing with peaks that do not meet the intensity difference requirement.

### 5.2. Peak refinement and box fitting

The 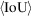 value in Table 3 ▸ gives the intersection between the labelled and detected peak positions. We deem the values of 49 and 35% not good enough for subsequent Bragg peak analysis, such as intensity determination. We believe these values can be partially attributed to the ambiguity of the peak position determination in the azimuthal direction. The azimuthal intensity distribution is not always peaked but may be more complex. Therefore it is not always possible to determine a reliable peak position. Consequently, the automated solutions encountered difficulties with it. While both models leave room for improvement, the Faster R-CNN, trained on a diverse range of peak shapes, demonstrates superior capability in the azimuthal position determination.

In contrast, 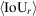 in the radial direction exhibits significantly better results with the value of 64%. The models can fit the radial shape accurately due to the Gaussian-like shape of the peaks and ring segments, which reduces ambiguity. The accurate determination of the radial peak position is crucial since it directly determines the uncertainty of lattice parameter estimation. The median shows similar results for both methods of 6.24 × 10^−3^ and 6.26 × 10^−3^ Å^−1^. However, the modified Faster R-CNN outperforms the region-growing approach in the 5th and 95th percentiles, achieving 0.47 × 10^−3^ and 17.76 × 10^−3^ Å^−1^ versus 0.83 × 10^−3^ and 24.25 × 10^−3^ Å^−1^. The values for both approaches are deemed acceptable for further processing such as structure identification.

### 5.3. Real time analysis

Modern X-ray sources allow measurement of X-ray scattering patterns at frequencies from a few hertz to megahertz (Li *et al.*, 2024 ▸; Decking *et al.*, 2020 ▸; Buffet *et al.*, 2012 ▸). Manually analysing hundreds of images per second is unrealistic. Consequently, a conventional approach would involve selecting a single image and analysing it, which could take anywhere from minutes to several hours depending on the complexity of the patterns. In contrast, the high processing rate in automated data analysis unlocks new types of experiments such as closed-loop experiments (Pithan *et al.*, 2023 ▸).

The modified Faster R-CNN model evaluated here benefits from the optimized software packages that are readily available, leveraging massively parallel algorithms, which results in an impressive number of 122 images processed per second (Starostin *et al.*, 2022*a* ▸). This represents a significant acceleration when contrasted with the sequential region-growing approach, which handles approximately one image per second.

## 6. Conclusions

We have established a comprehensive benchmark for automated Bragg peak detection in the GIWAXS geometry. We have provided a labelled dataset, which spans a wide spectrum of practically relevant scenarios. We have proposed a new metric tailored to the specifics of the GIWAXS data that focus on physically relevant aspects of the detection performance. It is well adapted for Bragg peak detection, emphasizing the overlap in the radial direction instead of employing plain IoU. As a baseline, we developed a region-growing algorithm that was fine-tuned on this dataset. Our framework based on a recent DL method exhibits superior performance compared with this classical baseline fine-tuned on the test dataset. The findings indicate that the DL-based approach particularly excels at identifying the peak positions and boundaries. Noteworthy advantages of the DL method lie in accurately determining the azimuthal profile. Detecting low-intensity peaks is challenging due to the complexity of differentiating them from the background. Furthermore, identifying adjacent peaks poses difficulties, particularly in the context of non-maximum suppression used for the Faster R-CNN. Future work could aim to enhance the simulation or incorporate experimental data for training purposes. Furthermore, DL architectures that do not use non-maximum suppression could prove beneficial.

## Supplementary Material

Supporting information file. DOI: 10.1107/S1600576725000974/vb5088sup1.pdf

Labeled GIWAXS dataset: https://doi.org/10.5281/zenodo.11545913

## Figures and Tables

**Table 1:** Tunable parameters of region-growing approach

Parameter	Value
Size of input image	1024 × 512 pixels
Filters used for image smoothing	Gaussian filter with kernel size 3 × 25
	Box filter with kernel size 3 × 3
Kernel size of maximum filter	3 × 3 pixels
Threshold for region-growing algorithm	5.5% of image intensity
Threshold for fit in azimuthal direction	5.5% of image intensity
Fitting function in radial direction	Gaussian function

**Table 2:** Classification of detection results based on the IoU criterion

True positive (TP)	Detection successful: IoU threshold for intersection with ground truth box is met
False positive (FP)	Detected box has not met an IoU intersection threshold with a ground truth box
False negative (FN)	No box found meets the IoU threshold of the ground-truth box

**Table 3:** GIWAXS-specific metrics for the peak-detection results in the composed dataset Better results are marked in bold.

Metric	Modified Faster R-CNN			Region-growing approach		
Confidence	High	Medium	Low	High	Medium	Low
Recall (%)	**95**	**83**	55	88	73	**60**

*AP_r_* (%)	70			–		
*P_r_* (%)	**87**			62		
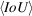 (%)	**49**			35		
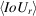 (%)	64			64		

Percentile	5	50	95	5	50	95
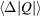 (10^−3^ Å^−1^)	**0.47**	**6.24**	**17.76**	0.83	6.26	24.25
