# Supplementary material for: Benchmarking deep learning for automated peak detection on GIWAXS data
Source: J Appl Crystallogr. 2025 Feb 28;58(Pt 2):513–22. doi: 10.1107/S1600576725000974 (PMC11957406; doi:10.1107/S1600576725000974)
Supplement: Supplementary file 1 [file j-58-00513-sup1.pdf]

JOURNAL OF
APPLIED
CRYSTALLOGRAPHY

Volume 58 (2025)

Supporting information for article:

Benchmarking deep learning for automated peak detection on GIWAXS data

Constantin Völter, Vladimir Starostin, Dmitry Lapkin, Valentin Munteanu, Mikhail Romodin, Maik Hylinski, Alexander Gerlach, Alexander Hinderhofer and Frank Schreiber

Appendix A
List of structures

Table 1. *List of structures used for dataset in section 2. The uncommon abbreviations are defined in Table 2*

**Sample structure**	**Perovskite type**
SiO_2|ITO|$FAPbI_3$	3D
SiO_2|ITO|$FAPbBr_3$	3D
SiO_2|ITO|$FAPbI_{1.2}Br_{1.8}$	3D
SiO_2|ITO|$MAPbI_3$	3D
SiO_2|ITO|$MAPbI_{1.2}Br_{1.8}$	3D
SiO_2|ITO|$MAPbI_{1.8}Br_{1.2}$	3D
SiO_2|ITO|$MAPbI_3$	3D
SiO_2|FTO|TiO_2|$FA_{.79}MA_{.16}Cs_{.05}PbI_{.85}Br_{.15}$|CuSCN	3D
SiO_2|FTO|TiO_2|$FAPbI_3$	3D
SiO_2|FTO|TiO_2|$MAPbBr_3$	3D
SiO_2|FTO|TiO_2|$FAPbI_3$	3D
SiO_2|ITO|$(BA)_2PbI_4$	2D, RP ($n = 1$)
SiO_2|ITO|$(BA)_2MA_2Pb_3I_{10}$	2D, RP ($n = 3$)
SiO_2|ITO|$(PEA)_2MA_2Pb_3I_{10}$	2D, RP ($n = 3$)
SiO_2|ITO|$(BA)_2MAPb_2I_7$	2D, RP ($n = 2$)
SiO_2|FTO|TiO_2|$(PDMA)FAPb_2I_7$	2D, DJ ($n = 2$)
SiO_2|FTO|TiO_2|$(PDMA)FA_3Pb_4I_{13}$	2D, DJ ($n = 4$)
SiO_2|FTO|TiO_2|$(PDMA)FA_2Pb_3I_{10}$	2D, DJ ($n = 3$)
SiO_2|FTO|TiO_2|$(PDMA)PbI_4$	2D, DJ ($n = 1$)
SiO_2|FTO|TiO_2|$(ADAM)_2PbI_4$	2D, RP ($n = 1$)
SiO_2|FTO|TiO_2|$(ADA)_2FA_2Pb_3I_7$	2D, RP ($n = 3$)
SiO_2|FTO|TiO_2|$(ADAM)_2FA_2Pb_3I_{10}$	2D, RP ($n = 3$)
SiO_2|FTO|TiO_2|$(ADAM)_2FA_3Pb_4I_{13}$	2D, RP ($n = 4$)
SiO_2|InO_x	–
SiO_2|CuSCN	–
SiO_2|FTO|TiO_2|$PbBr_2$	–
SiO_2|FTO|TiO_2|PbI	–

Table 2. *List of abbrevations used in Table 1*

RP	Ruddlesden–Popper
DJ	Dion–Jacobson
MA	Methylammonium (CH_3NH_3)
FA	Formamidinium ($CH(NH_2)_2$)
BA	Butylammonium($C_4H_9NH_3$)
PEA	Phenylethylammonium ($C_8H_{11}N$)
ADAM	(1-adamantyl)methanammonium(C_{11}H20N)
ADA	(1-adamantyl)ammonium($C_{10}H_{18}N$))
PDMA	Phenylenedimethanammonium ($C_8H_{12}N_2$)
FTO	Fluorine-doped tin oxide (SnO_2/F)
ITO	Indium tin oxide ($In_{18}O_{29}Sn$)

Appendix B
Region-growing algorithm

Require:
  Image with Bragg peaks
  Intensity threshold
Ensure:
  procedure DetectPeaks(Image, Local Maxima, IntensityThreshold)
    Initialize an empty list for detected peaks.
    Start a flood-fill algorithm from the global maximum:
    while next local maximum exists **do**
      Traverse to the next maximum.
      if greater or equal maximum encountered **then**
        Discard the current maximum.
      else if next maximum is lower but within intensity threshold **then**
        Mark next maximum as listed.
      end if
    end while
    for all listed peaks **do**
      if peak is within valid peak region (within intensity threshold of highest peak) **then**
        Mark peak as processed. Delete from listed peaks.
      else
        Consider peak for next iterations.
      end if
    end for
    for all peak regions with multiple local maxima **do**
      Calculate the optimum maximum using minimum distance criterion.
      Add optimum maxima to the list of detected peaks.
      Return the list of detected peaks.
    end for
  end procedure
